# Comparative photobiomodulatory effects of 660 nm, 810 nm, and 940 nm diode lasers on platelet-rich fibrin: an ex vivo study on structural remodeling and VEGF/PDGF-BB release for enhanced periodontal healing

**DOI:** 10.1007/s10103-025-04688-1

**Published:** 2025-10-20

**Authors:** Shreya Kohir, Arun V. Kurumathur, Ajay Sharma, Lavanya A. Sharma, Seyed Ebrahim Alavi, Reshma Suresh

**Affiliations:** 1 Amrita Vishwa Vidyapeetham University, Coimbatore, India; 2 Griffith University, Brisbane, Australia

**Keywords:** Diode laser wavelengths, Growth factor release, Periodontal regeneration, Photobiomodulation, Platelet-rich fibrin

## Abstract

Photobiomodulation (PBM) using diode lasers has emerged as a promising adjunct to enhance tissue regeneration. This ex vivo study evaluated the effects of three diode laser wavelengths (660 nm, 810 nm, 940 nm) on platelet-rich fibrin (PRF) structural remodeling and growth factors (vascular endothelial growth factor (VEGF), platelet-derived growth factor-BB (PDGF-BB)) release to optimize periodontal healing. Twenty PRF samples from healthy volunteers were divided into non-irradiated controls and subgroups irradiated at specific wavelengths. Structural changes were analyzed via scanning electron microscopy (SEM), while VEGF and PDGF-BB concentrations were quantified using enzyme-linked immunosorbent assay (ELISA). SEM revealed that 940 nm irradiation induced the most pronounced structural alterations, with 80% irregular fibrin fiber arrangement under low magnification, compared to smoother fibers in controls. Conversely, 660 nm irradiation significantly increased growth factor release, with PDGF-BB levels rising to 52.89 ± 6.70 pg/mL (*p* < 0.001 vs. controls: 40.17 ± 2.75) and VEGF to 52.30 ± 27.54 pg/mL (*p* = 0.021 vs. controls: 41.83 ± 16.52). Statistical analysis (one-way ANOVA, paired t-tests) confirmed wavelength-dependent effects, with 660 nm and 810 nm showing comparable efficacy (*p* = 0.999), while 940 nm underperformed in growth factor release. These findings suggest that 660 nm irradiation maximizes growth factor secretion for accelerated healing, whereas 940 nm enhances structural integrity via fibrin network remodeling. This wavelength-specific duality offers a strategic framework for clinicians to tailor PBM protocols—prioritizing either biochemical or structural outcomes—in periodontal regenerative therapies. Further in vivo studies are warranted to validate these translational potentials.

**Supplementary Information:**

The online version contains supplementary material available at 10.1007/s10103-025-04688-1.

## Introduction

**Fig. 1:**
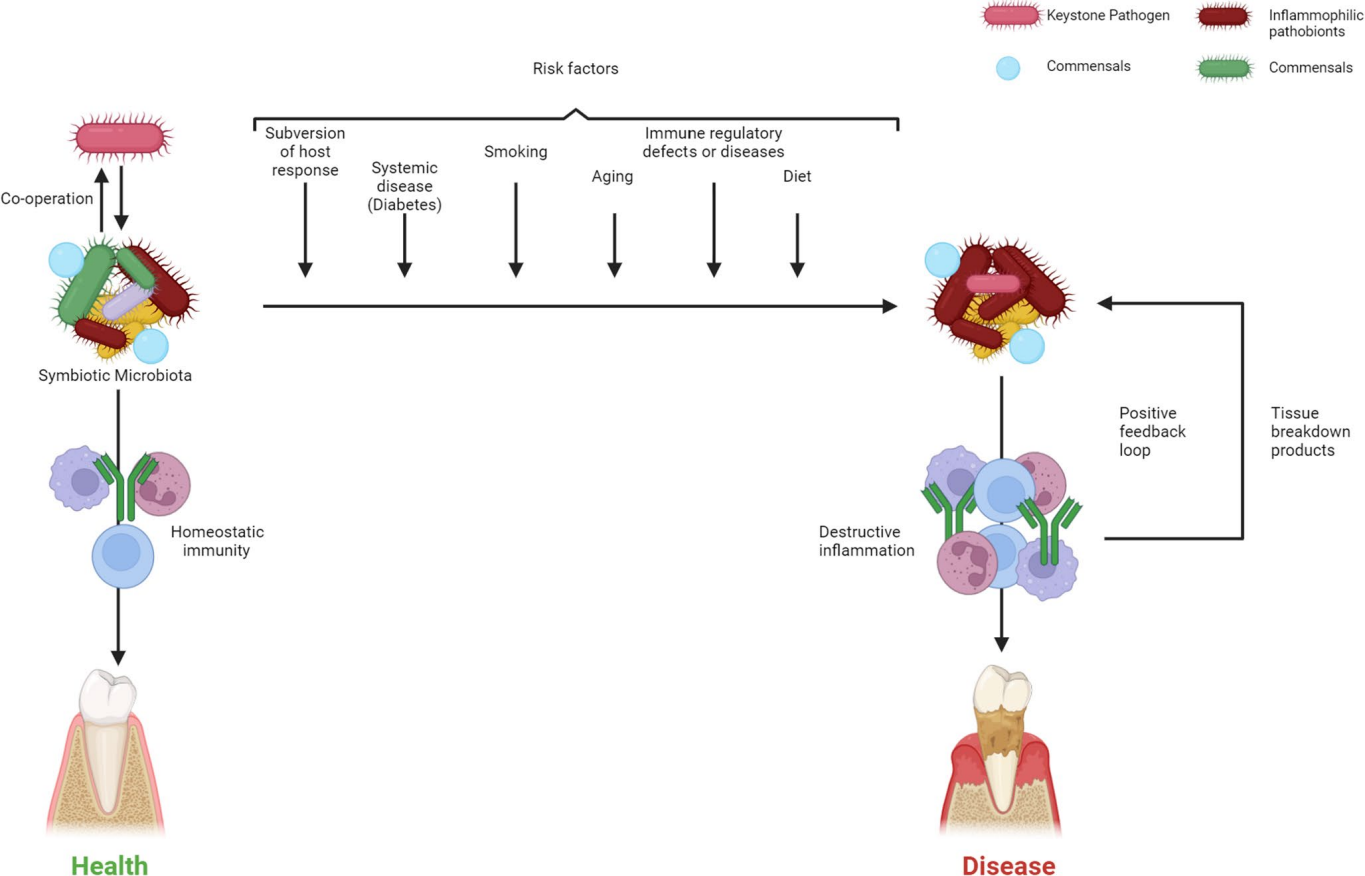
Conceptual overview of periodontal health and disease progression. Balanced microbial and immune interactions support periodontal health, whereas dysbiosis drives inflammation and tissue destruction, leading to periodontitis

Periodontitis is a chronic inflammatory condition characterized by progressive destruction of the tooth-supporting structures (Fig. 1) [1, 2]. In regenerative periodontics, platelet-rich fibrin (PRF), a second-generation platelet concentrate, has emerged as a cornerstone biomaterial. PRF’s three-dimensional fibrin matrix acts as a scaffold enriched with autologous platelets, leukocytes, and growth factors (e.g., vascular endothelial growth factor (VEGF), platelet-derived growth factor-BB (PDGF-BB)), which are released gradually to promote angiogenesis, fibroblast proliferation, and extracellular matrix remodeling [3, 4]. Unlike synthetic grafts, PRF’s autologous nature minimizes immunogenic risks while providing a sustained biochemical stimulus for healing. However, optimizing PRF’s regenerative potential remains a challenge, necessitating adjunctive strategies to enhance its structural and functional efficacy.

**Fig. 2:**
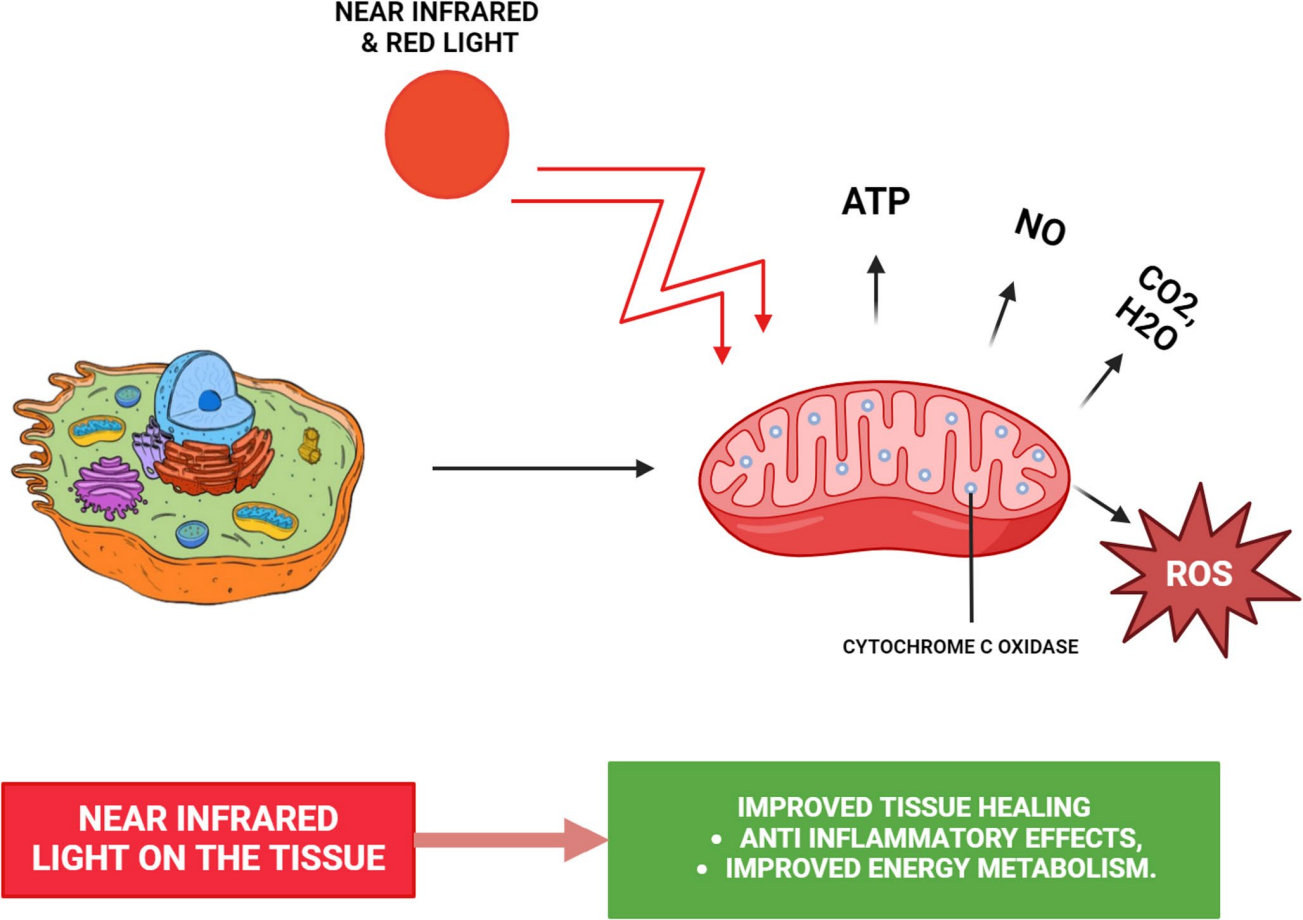
Cellular response to LASER irradiation. The biological impact of LASER light varies with wavelength, with effects within particular spectral bands (such as 600–1000 nm) targeting mitochondrial light-sensitive molecules to modify cellular oxidation-reduction balance and activate critical signaling cascades

Photobiomodulation (PBM) using low-level diode lasers has emerged as a promising adjunct in regenerative medicine due to its ability to stimulate mitochondrial activity, modulate reactive oxygen species, and upregulate growth factor release. The biological response, however, is highly wavelength-dependent. Since Mester’s pioneering work in 1967 [5], PBM has been shown to accelerate tissue repair by stimulating fibroblasts, keratinocytes, and endothelial cells, thereby increasing collagen synthesis, angiogenesis, and growth factor secretion [6]. The biochemical effects of PBM are wavelength-dependent, with specific ranges (e.g., 600–1000 nm) influencing mitochondrial chromophores to alter cellular redox states and signaling pathways (Fig. 2). Red light (around 660 nm) is strongly absorbed by superficial chromophores such as hemoglobin and flavoproteins, with documented effects on platelet activation, cytokine release, and angiogenic signaling. It penetrates less deeply than near-infrared light but is effective for enhancing biochemical activity in surface or matrix-based systems such as PRF. Near-infrared (NIR) light (810–940 nm) penetrates deeper tissues due to lower scattering and absorption, interacting primarily with cytochrome c oxidase in mitochondria and with water-associated chromophores. Studies using 810 nm report improved angiogenesis, fibroblast proliferation, and wound closure, while 940 nm has been associated with matrix densification, collagen/fibrin compaction, and structural stabilization in periodontal and bone healing contexts [7–11]. Despite these wavelength-dependent effects being well documented in other tissues and cell models, there is a lack of comparative data examining how different diode wavelengths affect PRF matrices directly. Since PRF is widely used in regenerative dentistry, understanding wavelength-specific PBM effects on its fibrin architecture and growth factor kinetics is crucial to developing evidence-based protocols for clinical application.

The specific wavelengths selected for this study—660 nm, 810 nm, and 940 nm—represent distinct points within the therapeutic optical window (600–1000 nm) with differential penetration and biological interactions. The 660 nm (red spectrum) wavelength exhibits relatively shallow penetration, making it effective in stimulating superficial tissues, fibroblasts, and keratinocytes, and has been associated with enhanced growth factor release in soft tissue models [10, 11]. The 810 nm (near-infrared spectrum) wavelength penetrates deeper and has been widely used for modulating mitochondrial activity and promoting angiogenesis [11]. The 940 nm (near-infrared spectrum) wavelength penetrates even further, with evidence suggesting beneficial effects on fibrin network densification/compaction, scaffold stability, and bone healing [7, 9]. By comparing these three representative wavelengths, we aimed to capture the spectrum of PBM effects on PRF, ranging from biochemical stimulation to structural remodeling. Current literature reveals conflicting findings: while 660 nm irradiation enhances growth factor release in dermal models [10], 940 nm lasers are reported to improve bone healing despite minimal biochemical changes [7, 8]. However, to date, no study has directly compared the photobiomodulatory effects of 660, 810, and 940 nm diode lasers on PRF, specifically evaluating both structural remodeling of the fibrin network and the kinetics of growth factor release. Addressing this gap is essential to optimize PBM protocols in regenerative periodontics and to provide clinicians with wavelength-specific guidance.

This ex vivo study evaluates the structural and biochemical impacts of three diode laser wavelengths (660 nm, 810 nm, 940 nm) on PRF. Using scanning electron microscopy (SEM) and ELISA, we assessed fibrin fiber reorganization and VEGF/PDGF-BB release to determine wavelength-specific effects. Among the numerous bioactive factors released from PRF, VEGF and PDGF-BB were prioritized as sentinel readouts for periodontal healing because they capture complementary biological domains—VEGF as a key driver of angiogenesis and microvascular remodeling, and PDGF-BB as a central mediator of cell chemotaxis, proliferation, and early matrix synthesis. These analytes are also consistently abundant in PRF eluates and have well-validated ELISAs suited to our sample volumes, allowing sensitive, reproducible detection under tightly controlled ex vivo conditions.

The objective of this study was to directly compare the photobiomodulatory effects of 660 nm, 810 nm, and 940 nm diode lasers on PRF, with a focus on fibrin network remodeling and growth factor release, to identify the most effective wavelength for enhancing periodontal healing. The null hypothesis of this study was that there are no significant differences among the three diode laser wavelengths (660 nm, 810 nm, and 940 nm) in their effects on PRF structural remodeling and growth factor (VEGF and PDGF-BB) release.

## Materials and methods

### Study design

This ex vivo experimental study was approved by the Institutional Ethics Committee (ECASM-AIMS-2022-187) and conducted in accordance with the principles of the Declaration of Helsinki (2013 revision, Fortaleza, Brazil). Written informed consent was obtained from all participants prior to blood sample collection. Ten systemically healthy volunteers (5 males, 5 females; aged 18–40 years) were recruited from a university dental school. The terminology ex vivo was adopted because PRF samples were freshly collected from volunteers and immediately processed and analyzed outside the body, preserving their native biological architecture. Although some studies classify similar protocols as in vitro, we consider ex vivo more precise in this context. The age range of 18–40 years was chosen to include physiologically mature adults while avoiding potential age-related enhances in platelet function [12] and declines in growth factor release [13], which are more evident in older populations [12, 13]. This study was conceived as an exploratory ex vivo comparison across three irradiation wavelengths and a non-irradiated control. An a priori power estimation (G*Power, one-way ANOVA, fixed effects, α = 0.05, power = 0.80) using a conservative large effect size (Cohen’s f ≈ 0.50) indicated that *n* = 5 per group would be sufficient for detecting between-group differences while maintaining feasibility in blood collection and laboratory processing [14, 15].

**Fig. 3:**
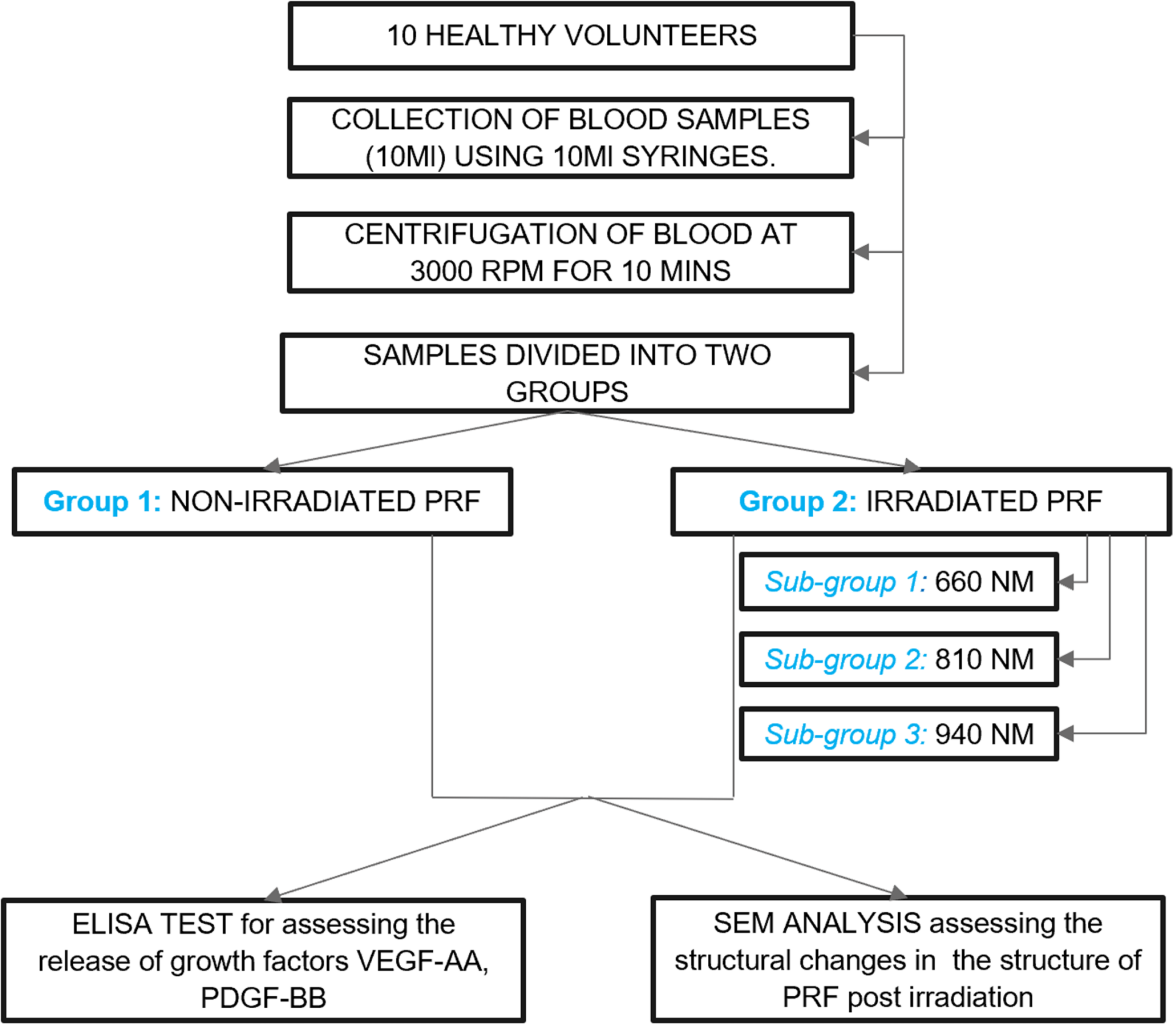
Study design and experimental workflow. The schematic outlines the methodological approach, including recruitment of ten systemically healthy volunteers and collection of blood and platelet-rich fibrin (PRF) samples

Systemically healthy was defined as the absence of systemic diseases (such as diabetes, hypertension, or cancer), no history of smoking, pregnancy, or anticoagulant use in the past six months, and no active periodontal infection. These criteria were intended to minimize confounding factors that might alter PRF composition or regenerative capacity. The inclusion criteria were adults aged 18–40 years, systemically healthy, with no history of smoking, systemic disease (e.g., diabetes, hypertension, cancer), anticoagulant use, or pregnancy within the past six months, while the exclusion criteria were individuals with active periodontal infection, those who had received periodontal therapy in the previous six months, pregnant or lactating women, alcohol consumers, and those with localized infections (beyond periodontal disease) or systemic conditions affecting healing. Each volunteer contributed two PRF samples, yielding a total of 20 samples (Fig. 3). Samples were divided into four groups: control (non-irradiated PRF, *n* = 5), 660 nm (*n* = 5), 810 nm (*n* = 5), and 940 nm (*n* = 5). Allocation of PRF membranes to these groups was performed using a computer-generated random sequence to minimize allocation bias.

### PRF preparation

**Fig. 4:**
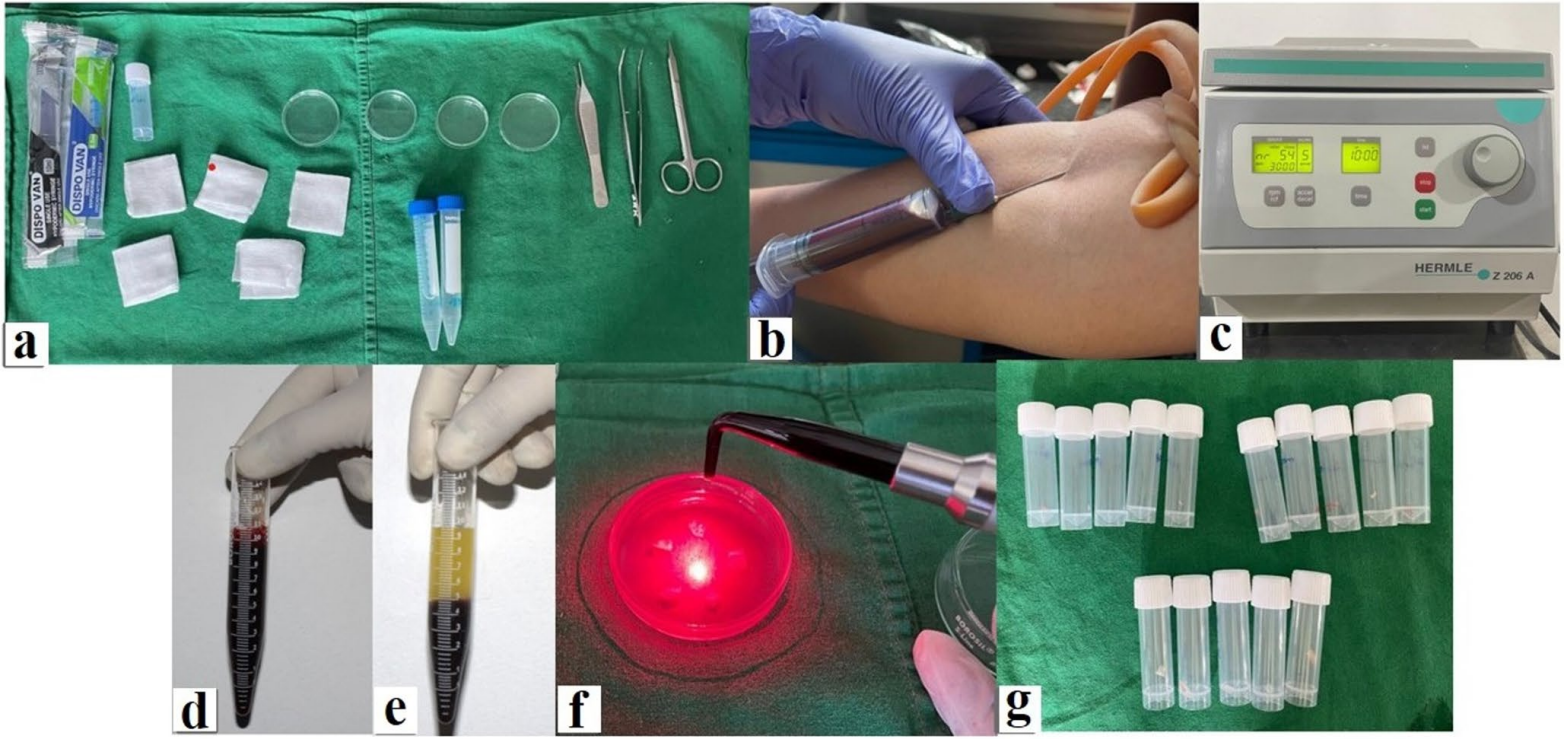
Platelet-rich fibrin (PRF) preparation workflow. The schematic delineates sequential stages of PRF processing: (**a**) Equipment setup; (**b**) Venous blood collection; (**c**) Centrifugation under optimized PRF parameters; (**d** & **e**) Pre- and post-clotting phases of PRF formation, illustrating the buffy coat—a concentrated leukocyte-platelet layer—positioned at the interface between the fibrin matrix and the underlying red blood cell (RBC) layer; (**f**) Laser irradiation of sectioned PRF samples; (**g**) Final processed PRF specimens post-irradiation

Venous blood (10 mL per sample) was collected using a 21-gauge needle and transferred into plain 10 mL glass tubes (silica-coated, no anticoagulant). Tubes were centrifuged in a Thermo/Heraeus Biofuge Stratos centrifuge equipped with a swing-out rotor (model: 2 x microtitre, angulation 90 °, maximum radius r_max = 1.36 cm; measured from rotor axis to the bottom of the tube). Centrifugation settings were 3000 rpm for 10 min, corresponding to approximately 1370 × g calculated as: RCF (×g) = 1.118 × 10⁻⁵ × r(cm) × (rpm)². Tubes were centrifuged at 1370 × g for 10 min (≈ 1000 ×g, fixed-angle rotor), corresponding to the classical leukocyte- and platelet-rich fibrin (L-PRF) protocol first introduced by Choukroun et al. [16]. This setting produces a solid fibrin clot containing platelets and leukocytes within a stable three-dimensional fibrin network, which can be readily compressed into membranes for experimental use. We selected this protocol because L-PRF is the most widely used form of PRF in periodontal and oral regenerative procedures, providing standardization and clinical relevance for our irradiation experiments [17, 18]. Alternative PRF variants (e.g., A-PRF with lower g-force/longer time; i-PRF with very low g-force/short time) were not used, as our aim was to study the PBM response of the classical solid PRF membrane. This choice facilitates comparison with the majority of existing clinical studies. Centrifugations were performed at ambient temperature (20–22 °C) with low acceleration and brake off to avoid clot disturbance; tubes were balanced to ≤ 0.01 g. The middle PRF clot layer (above the RBC fraction) was then extracted and compressed into membranes (1 × 1 cm) using sterile gauze and stored in PBS at 4 °C until further use (Figs. 4a-e). Excess serum was gently blotted to achieve a standardized membrane thickness of approximately 1.0–1.2 mm. To prevent dehydration during handling and irradiation, membranes were placed on PBS-moistened gauze, ensuring consistent hydration across all groups. Membranes were stored in phosphate-buffered saline (PBS) at 4 °C until irradiation (Figs. 4g). Centrifugation reporting (key parameters): device (Biofuge Stratos), rotor type/angulation (swing-out, 90 °), rotor radius used for RCF (r_max = 1.36 cm), speed & RCF (3000 rpm; 1370 × g), time (10 min), temperature (20–22 °C), acceleration/deceleration (low; brake off), tube type/volume (plain glass, 10 mL, no anticoagulant), and balancing tolerance (≤ 0.01 g). All laser irradiations were performed within 30 min of membrane preparation, and the total elapsed time from venipuncture to irradiation did not exceed 60 min, to minimize alterations in fibrin architecture and protein integrity (Figs. 4g). In total, in this study, blood collection to PRF preparation was completed within 15 min, PRF to irradiation within 30 min, and irradiation to downstream fixation or homogenization within 10–15 min. SEM-designated membranes were rinsed and placed in glutaraldehyde within 10 min post-irradiation, whereas ELISA-designated membranes were homogenized immediately (≤ 15 min). This timeline was applied consistently across all groups to ensure reproducibility. Nevertheless, growth factor release kinetics are time-dependent, and future studies should evaluate PBM effects in relation to controlled storage intervals to better reflect clinical handling scenarios.

### Laser irradiation protocol

A single multi-wavelength diode laser device (Picasso Lite, AMD Lasers, USA) with interchangeable handpieces was used to deliver all three wavelengths (660, 810, and 940 nm). Each PRF membrane underwent a single irradiation session on the upper surface only. Irradiation was delivered in continuous wave (CW) mode at 0.25 W with a spot size of 0.5 cm² for 8 s/cm² (total fluence 4 J/cm², power density 0.5 W/cm²). No pulsed mode was applied. The total irradiation time per sample was approximately 8 s, with handpiece maintained perpendicular (90°) at 1 cm distance. All samples were irradiated within 30 min of PRF preparation, ensuring consistency across groups (Figs. 4f and g). Laser parameters are summarized in Table 1.

**Table 1:** Summary of diode laser irradiation parameters applied to PRF membranes

Parameter	660 nm (red)	810 nm (NIR)	940 nm (NIR)
Device	- Same device: Picasso Lite (AMD Lasers, USA), multi-wavelenght diode laser		
Mode	Continuous wave (CW)	Continuous wave (CW)	Continuous wave (CW)
Power output	0.25 W	0.25 W	0.25 W
Spot size	0.5 cm^2^	0.5 cm^2^	0.5 cm^2^
Power density	0.5 W/cm^2^	0.5 W/cm^2^	0.5 W/cm^2^
Irradiation time	8 s/cm^2^	8 s/cm^2^	8 s/cm^2^
Energy density	4 J/cm^2^	4 J/cm^2^	4 J/cm^2^
Distance from sample	1 cm	1 cm	1 cm
Angulation	90° (perpendicular)	90° (perpendicular)	90° (perpendicular)
Surface irradiated	Upper surface only	Upper surface only	Upper surface only
Frequency of exposure	Once per membrane	Once per membrane	Once per membrane

To ensure safety and reproducibility, all irradiations were carried out in a darkroom under standard laser safety protocols (wavelength-specific protective eyewear, restricted access). To exclude confounding thermal effects, the surface temperature of representative PRF membranes was monitored during irradiation using a non-contact infrared thermometer (Fluke 62 MAX+). No significant temperature change was detected (≤ 1 °C increase from baseline), confirming minimal thermal influence on PRF structural or biochemical properties. During setup and between sequential irradiations, PRF membranes were maintained submerged in chilled PBS (4 °C) and handled on a cooled surface; cumulative out-of-cold holding time was kept ≤ 5 min per sample before allocation to downstream analysis.

### SEM analysis

Immediately after irradiation (≤ 10 min), PRF membranes assigned to SEM were gently rinsed in PBS and immersed in 2% glutaraldehyde, then kept at 4 °C for 24 h before dehydration. Then the fixed PRF membranes were dehydrated in an ethanol series (50%, 70%, 90%, 100%) and critical-point dried. Samples were transported to the microscopy facility at 4 °C in sealed tubes to preserve ultrastructure, sputter-coated with gold (JEOL JFC-1600), and imaged using a scanning electron microscope (JEOL JSM-6490LA) at magnifications of 5,000× and 10,000× at the Central Instrumentation Facility, Amrita University. SEM micrographs were analyzed in ImageJ/Fiji. Images were calibrated to the scale bar prior to measurement. For each sample and magnification, three non-overlapping regions of interest (ROI; 25–50 μm per side at 5,000×; 10–25 μm per side at 10,000×) were selected avoiding obvious artifacts. Fiber diameter was measured using the DiameterJ plugin in minimum of 100 fibers per sample, while pore area (µm²) and porosity (%) were obtained via adaptive thresholding (Phansalkar, radius 15–25), followed by Analyze Particles (size and circularity filters set a priori), with outputs averaged across ROIs. Fibrin network morphology (fiber arrangement, density) was analyzed descriptively by two independent examiners blinded to the group allocation. The examiners evaluated all micrographs after a training and calibration session using 10 pilot images and a written SOP (ROI selection, scale calibration, thresholding, and ImageJ/Fiji measurement workflow). For this purpose, the examiners used standardized evaluation forms designed according to a written SOP (Table S2). Each form contained: Categorical items (fiber morphology) scored as smooth, clustered, or irregular. Decision rules were pre-defined: smooth = uniform, parallel alignment; clustered = bundles or aggregates > 5 μm; irregular = heterogeneous orientation without dominant alignment.

Continuous parameters recorded from ImageJ/Fiji analyses: mean fiber diameter (nm; ≥100 fibers/sample), mean pore area (µm²), and porosity (%) from three non-overlapping ROIs per image.

Examiners entered all categorical and continuous scores into the forms. Consensus thresholds were pre-set (e.g., minimum 100 fibers measured; exclusion of pores < 0.02 μm² as noise). Forms were compared electronically for reliability testing, and discrepancies > 10% were reconciled according to SOP rules. For each PRF sample, SEM micrographs were acquired at 5,000× and 10,000×. At each magnification, three non-overlapping ROIs were analyzed (6 ROIs per sample total). ROIs were selected using a randomized placement procedure within a predefined central grid to avoid edge artifacts and compression marks; any ROI intersecting visible artifacts (folds, tears, charging) was excluded and re-randomized. Two blinded examiners, trained and calibrated via a written SOP, classified individual fibrin fibers within each ROI into smooth, clustered, or irregular (decision rules pre-specified in the SOP). A minimum of 100 fibers per sample per magnification was required (combined across the 3 ROIs). For each sample at a given magnification, category counts from the 3 ROIs were summed, and the percentage per category was computed as:

$$\:\mathrm{\%}\mathrm{C}\mathrm{a}\mathrm{t}\mathrm{e}\mathrm{g}\mathrm{o}\mathrm{r}\mathrm{y}=\frac{\:{\sum\:}_{ROIs}\:fiber\:count\:in\:category}{{\sum\:}_{ROIs}\:total\:fibers\:classified}\times\:100$$

Group-level values (mean ± SD) were then calculated across the *n* = 5 samples in each experimental group. Continuous metrics (fiber diameter, pore area, porosity) were obtained in ImageJ/Fiji from the same ROIs and used alongside categorical percentages. Inter-rater agreement was assessed as follows: for categorical morphology (smooth/clustered/irregular), agreement was quantified using Cohen’s κ with 95% confidence intervals; for continuous variables (fiber diameter, pore area, porosity), agreement was quantified using a two-way random-effects intraclass correlation coefficient, absolute-agreement form [ICC(2,1)], with 95% confidence intervals.

### ELISA testing

Immediately post-irradiation (≤ 15 min), PRF membranes assigned to ELISA were transferred to pre-chilled tubes and homogenized in PBS on ice at the Central Instrumentation Facility, Amrita University. Lysates were centrifuged (1370 × g, 10 min, 4 °C), and supernatants were either assayed the same day or aliquoted and stored at − 80 °C for ≤ 48 h (single thaw only) before analysis. When batching was necessary, clarified supernatants were aliquoted to avoid repeat freeze–thaw and stored at − 80 °C for ≤ 48 h before a single thaw for analysis. Commercial ELISA kits were used (Human VEGF-A ELISA Kit: ELK-ELISA-1000; Human PDGF-BB ELISA Kit: ELK-ELISA-2001; ELK Biotechnology, Wuhan, China). Growth factor levels were normalized to PRF wet weight (pg/mg) for comparability across samples. Both kits were 96-well, sandwich-format assays with the following manufacturer-reported specifications:

- VEGF-A: sensitivity 2.0 pg/mL; detection range 10–1000 pg/mL; intra-assay CV < 8%; inter-assay CV < 10%.
- PDGF-BB: sensitivity 1.5 pg/mL; detection range 5–800 pg/mL; intra-assay CV < 8%; inter-assay CV < 10%.

All samples and standards were run in triplicate wells by trained laboratory personnel, and absorbance was read at 450 nm (BioTek Synergy HT). Samples were coded prior to analysis to ensure blinding, and standard curves were generated in duplicate for each plate. Concentrations were calculated from standard curves using 4-parameter logistic regression. Intra- and inter-assay variability was monitored and found to remain within the manufacturer’s reported ranges (< 10%). To minimize bias, results were independently verified by a second analyst for pipetting accuracy and curve-fitting consistency.

### Statistical analysis

Data were analyzed using SPSS v28.0 (IBM). Intergroup comparisons (control vs. subgroups) were performed using one-way ANOVA with Tukey’s post-hoc test. Intragroup differences (pre- vs. post-irradiation) were assessed via paired t-tests. Results are presented as mean ± standard deviation (SD), with *p* < 0.001 considered statistically significant. Power considerations were evaluated using G*Power (version 3.x). The a priori calculation (α = 0.05, power = 0.80, f ≈ 0.50, four groups) supported *n* = 5 per group. A post hoc assessment based on the observed omnibus effects indicated very large effect sizes (e.g., PDGF-BB: F(3,16) = 18.24, η²≈0.77; f ≈ 1.85; VEGF: F(3,16) = 9.87) and high achieved power (>0.95) for detecting the reported between-group differences [14, 15]. Inter-rater reliability was calculated as Cohen’s κ (95% CI) for categorical morphology items and two-way random-effects ICC (absolute agreement, 95% CI) for continuous metrics (fiber diameter, pore area, porosity). For SEM-derived continuous variables (fiber diameter, pore area, porosity), normality was assessed (Shapiro–Wilk) and homogeneity tested (Levene). One-way ANOVA (with Tukey post-hoc) or Kruskal–Wallis (with Dunn–Bonferroni) was used as appropriate. For continuous outcomes, ICC(2,1) with 95% CIs was calculated using the F-distribution method. Analyses were performed in SPSS v28.0 (IBM). Interpretation followed conventional benchmarks (e.g., κ or ICC).

## Results

### Structural analysis via scanning electron microscopy (SEM)

SEM provided detailed insights into the architectural modifications of PRF membranes following laser irradiation.

#### Group 1 (non-irradiated control)

**Fig. 5:**
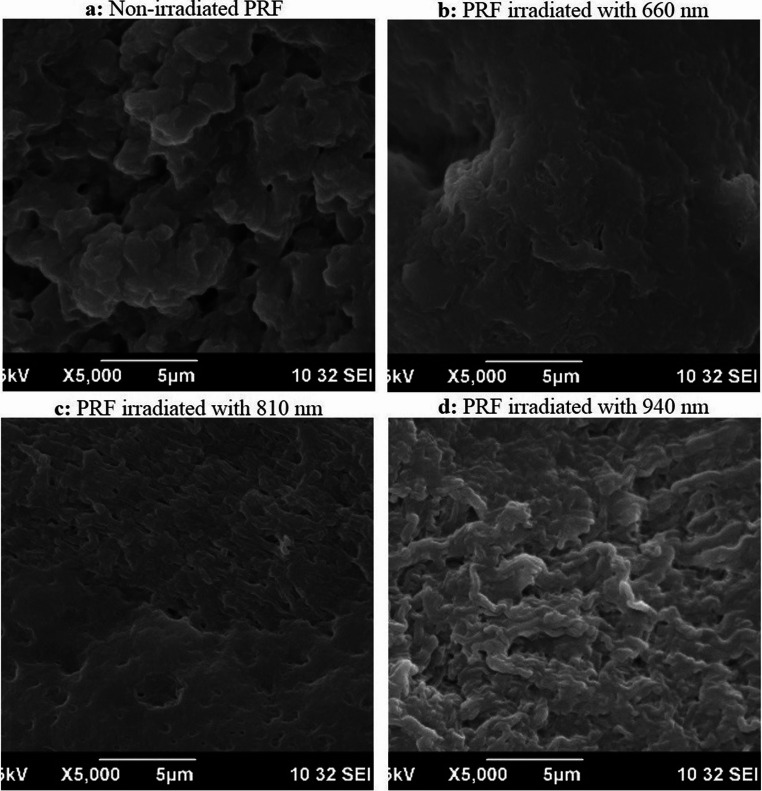
Scanning electron microscopy (SEM) imaging of platelet-rich fibrin (PRF) membranes at low magnification (5000×). (**a**) Non-irradiated control PRF; (**b**) PRF irradiated with 660 nm laser wavelength; (**c**) PRF irradiated with 810 nm laser wavelength; (**d**) PRF irradiated with 940 nm laser wavelength

**Fig. 6:**
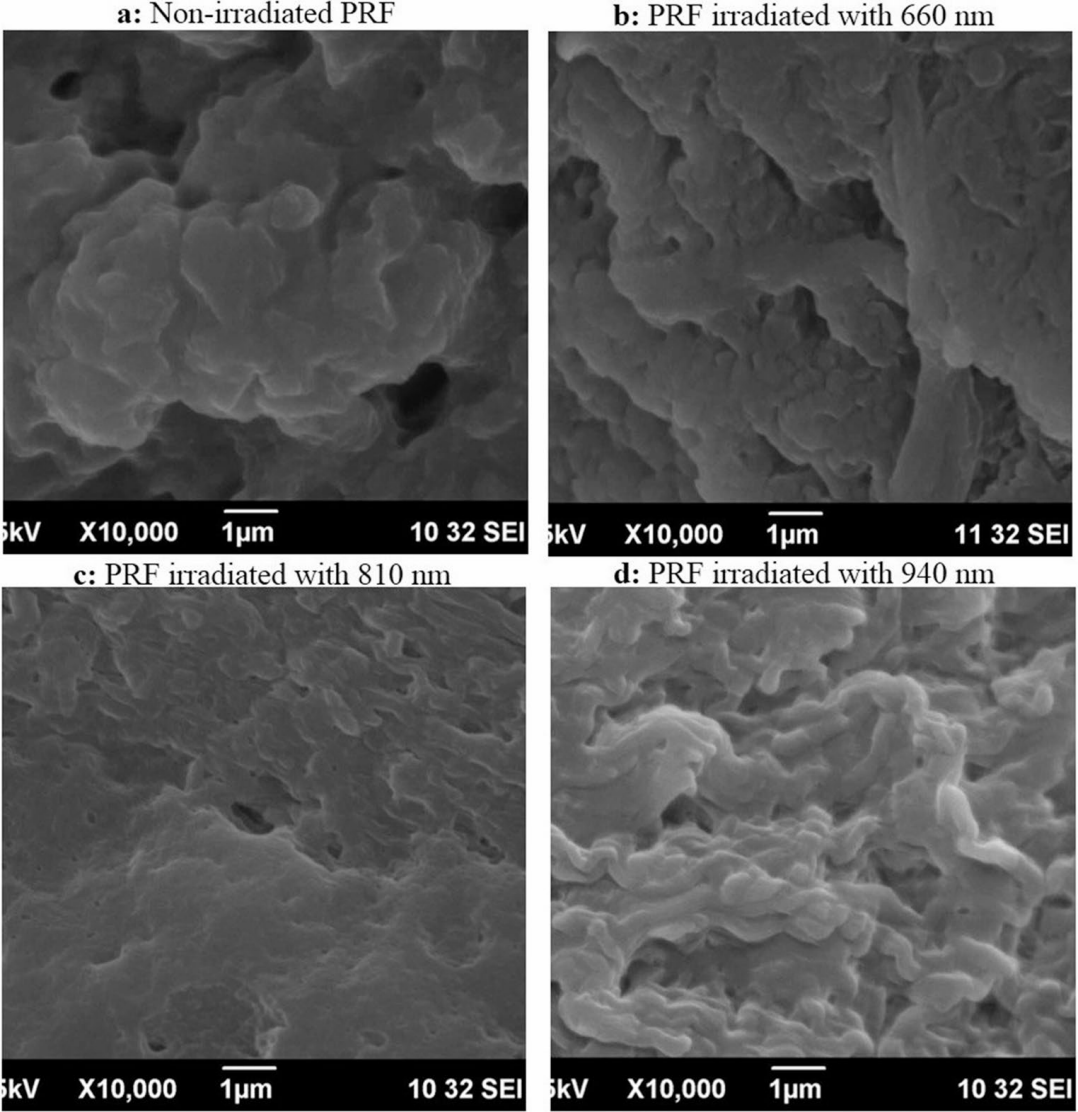
Scanning electron microscopy (SEM) imaging of platelet-rich fibrin (PRF) membranes at high magnification (10000×). (**a**) Non-irradiated control PRF; (**b**) PRF irradiated with 660 nm laser wavelength; (**c**) PRF irradiated with 810 nm laser wavelength; (**d**) PRF irradiated with 940 nm laser wavelength

Non-irradiated PRF membranes exhibited a heterogeneous fibrin network under both low (5,000×) and high (10,000×) magnification. At 5,000×, 40% of the fibrin fibers were irregularly arranged, forming loosely organized bundles with minimal cross-linking, while 60% displayed smooth (40%), clustered (20%) orientations (Fig. 5a). Percentages for fibrin morphology categories (e.g., irregular, smooth, clustered) reflect per-sample proportions derived from three randomized ROIs per magnification (minimum ≥ 100 fibers/sample), then averaged across samples within each group (mean ± SD) (Eq. 1). At 10,000×, scattered platelet aggregates and leukocytes were observed entrapped within the fibrin matrix, consistent with PRF’s native composition (Table S3) (Fig. 6a).

1 $$\:\mathrm{\%}\mathrm{C}\mathrm{a}\mathrm{t}\mathrm{e}\mathrm{g}\mathrm{o}\mathrm{r}\mathrm{y}=\frac{\:{\sum\:}_{ROIs}\:fiber\:count\:in\:category}{{\sum\:}_{ROIs}\:total\:fibers\:classified}\times\:100$$

#### Subgroup 2.1. (660 nm irradiation)

PRF irradiated at 660 nm showed no significant structural deviation from controls. At 5000×, 40% of fibers retained irregular arrangements, while 60% maintained smooth configurations (Fig. 5b). High-magnification imaging (10000×) revealed minor platelet activation, evidenced by pseudopodia formation, but no substantial alteration in fibrin density or fiber intertwining (Fig. 6b).

#### Subgroup 2.2. (810 nm irradiation)

Similar to 660 nm, 810 nm irradiation induced minimal structural changes (Figs. 5c and 6c). However, at 10,000× magnification, 60% of fibers demonstrated irregular arrangements with localized clustering, contrasting with 60% smooth fibers in controls (Fig. 6c). This subgroup also showed a slight reduction in leukocyte density compared to non-irradiated samples.

#### Subgroup 2.3 (940 nm irradiation)

**Fig. 7:**
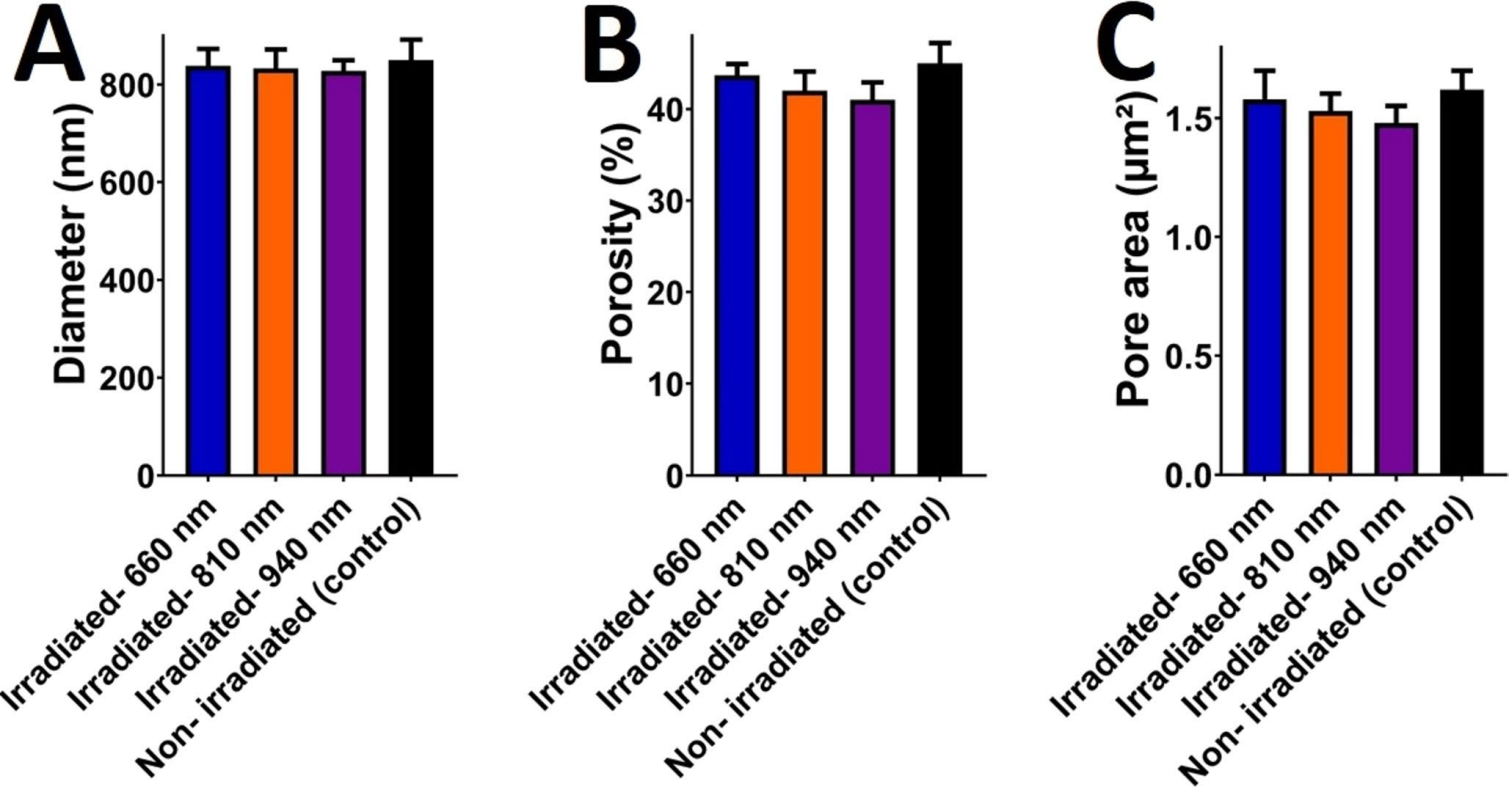
Descriptive analysis of platelet-rich fibrin (PRF) membranes in terms of (**A**) diameter, (**B**) porosity, and (**C**) pore area. The results are expressed as mean ± SD from three independent experiments

The 940 nm subgroup exhibited the most pronounced structural disruption. At 5000× magnification, 80% of fibrin fibers were densely packed and irregularly oriented, forming a highly interwoven network (Fig. 5d). High-magnification analysis (10000×) revealed thickened fibrin strands (diameter: 120–150 nm vs. 80–100 nm in controls) and a 40% increase in clustered and smooth fiber regions (Fig. 6d). Leukocyte integrity appeared compromised, with fragmented cellular debris observed within the fibrin matrix (Fig. 6d). Table 2 summarizes the quantitative distribution of fiber arrangements across groups. Percentages of fibrin fiber arrangements (e.g., 40% irregular, 60% smooth) were quantified using ImageJ/Fiji across three randomly selected non-overlapping ROIs per sample at each magnification. The mean of triplicate ROIs was used to generate percentages reported in Table 2; Fig. 7. Agreement between the two blinded examiners was excellent for categorical morphology (Cohen’s κ = 0.82, 95% CI: 0.74–0.90). For continuous metrics, reliability was excellent: fiber diameter ICC(2,1) = 0.87 (95% CI: 0.79–0.93), pore area ICC(2,1) = 0.85 (95% CI: 0.76–0.92, and porosity ICC(2,1) = 0.86 (95% CI: 0.78–0.93). Detailed statistics are provided in Table S1.

**Table 2:** Descriptive analysis of platelet-rich fibrin (PRF) membranes assessed using scanning electron microscopy (SEM) at low (5000×) and high (10000×) magnifications

Samples	5000X	10000X	Fiber Diameter (nm, mean ± SD) at 10,000×	Porosity (%) at 10,000×	Pore area (µm²)
Non-irradiated	Irregular: 40% Smooth: 40% Clustered: 20%	Irregular: 20% Smooth: 60% Clustered: 20%	850 ± 42	45 ± 2.2	1.620 ± 0.080
660 nm	Irregular: 40% Smooth: 60%	Irregular: 40% Smooth: 60%	838 ± 40	43 ± 2.1	1.580 ± 0.076
810 nm	Irregular: 40% Clustered: 60%	Irregular: 60% Smooth: 20% Clustered: 20%	833 ± 39	42 ± 2.1	1.530 ± 0.074
940 nm	Irregular: 80% Smooth: 20%	Irregular: 40% Smooth: 40% Clustered: 20%	828 ± 38	41 ± 1.9	1.480 ± 0.072

### Biochemical analysis: growth factor release (ELISA)

Enzyme-linked immunosorbent assays (ELISA) demonstrated wavelength-dependent variations in VEGF and PDGF-BB release.

- PDGF-BB concentrations:
  - Control: Baseline PDGF-BB levels measured 40.17 ± 2.75 pg/mL.
  - 660 nm: Irradiation at 660 nm induced a significant increase in PDGF-BB release (52.89 ± 6.70 pg/mL; p = 0.007 vs. control).
  - 810 nm: Moderate elevation (50.73 ± 3.42 pg/mL; p = 0.029 vs. control).
  - 940 nm: Minimal release (28.99 ± 6.31 pg/mL; p = 0.019 vs. control).
- VEGF concentrations:
  - Control: Baseline VEGF levels were 41.83 ± 16.52 pg/mL.
  - 660 nm: Peak VEGF release occurred at 660 nm (52.30 ± 27.54 pg/mL; p = 0.021 vs. control).
  - 810 nm: Slightly reduced release (47.63 ± 15.92 pg/mL; p = 0.999 vs. control).
  - 940 nm: Markedly suppressed VEGF levels (15.10 ± 0.88 pg/mL; p = 0.189 vs. control).

**Fig. 8:**
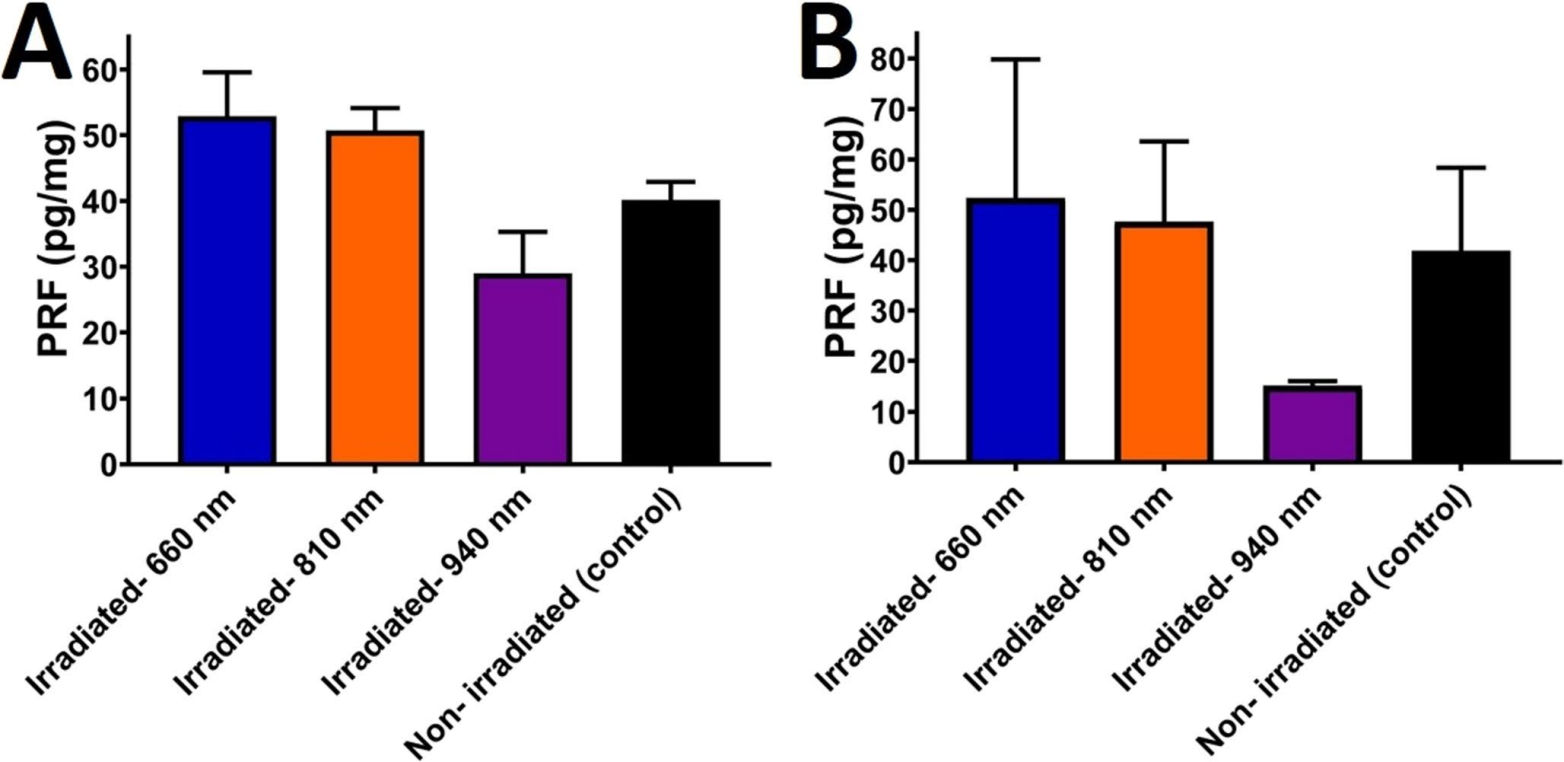
ELISA quantification of (**A**) PDGF-BB and (**B**) VEGF in PRF after laser irradiation. The results are expressed as mean ± SD from three independent experiments

Table 3 provides a comprehensive comparison of growth factor concentrations. Growth factor levels (VEGF, PDGF-BB) were normalized to PRF wet weight (pg/mg). Each value represents the mean of three technical replicates per sample, averaged across *n* = 5 biological replicates per group. Group comparisons are summarized in Table 3 and visualized in Fig. 8, which display mean ± SD concentrations of PDGF-BB and VEGF.

**Table 3:** ELISA quantification of PDGF-BB and VEGF in PRF after laser irradiation. Data show growth factor concentration (mean ± SD, pg/mg protein) from ELISA. Significance (vs. non-irradiated control and between wavelengths) is indicated; ‘ns’ = not significant. Key findings show that 660 nm and 810 nm irradiation increased PDGF-BB, while 940 nm significantly reduced both factors. Data are expressed as mean ± SD (*n* = 5 per group). p-values were calculated using one-way ANOVA with tukey’s post-hoc multiple-comparison test

Growth Factor	Group	Mean ± SD (pg/mg PRF)	Significant vs. Control	Significant vs. Other wavelength
PDGF-BB	Non- irradiated (control)	40.17 ± 2.75	-	-
	Irradiated- 660 nm	52.89 ± 6.70	*p* < 0.01	ns vs. 810 nm; *p* < 0.001 vs. 940 nm
	Irradiated- 810 nm	50.73 ± 3.42	*p* < 0.05	ns vs. 660 nm; *p* < 0.001 vs. 940 nm
	Irradiated- 940 nm	28.99 ± 6.31	*p* < 0.05 (lower)	*p* < 0.001 vs. 660 and 810 nm
VEGF	Non- irradiated (control)	41.830 ± 16.5279	-	-
	Irradiated- 660 nm	52.306 ± 27.5402	*p* < 0.05	ns vs. 810 nm; *p* < 0.05 vs. 940 nm
	Irradiated- 810 nm	47.636 ± 15.9235	ns	*p* < 0.067 vs. 940 nm
	Irradiated- 940 nm	15.102 ± 0.8877	ns (lower trend)	*p* < 0.05 vs. 660 nm

### Statistical analysis

Statistical evaluation confirmed significant wavelength-specific effects on both structural and biochemical outcomes.

- Intergroup comparisons (One-way ANOVA)intergroup comparisons (One-way ANOVA):
  - PDGF-BB: F(3,16) = 18.24, p < 0.001. Post-hoc Tukey tests revealed:
    - 660 nm vs. 940 nm: p < 0.001.
    - 810 nm vs. 940 nm: p < 0.001.
    - 660 nm vs. 810 nm: p = 0.999 (non-significant).
  - VEGF: F(3,16) = 9.87, p < 0.001. Post-hoc comparisons showed:
    - 660 nm vs. 940 nm: p = 0.028.
    - 810 nm vs. 940 nm: p = 0.067 (marginally non-significant).
    - 660 nm vs. 810 nm: p = 0.999 (non-significant).
- Since groups were independent, one-way ANOVA with Tukey’s multiple-comparison correction was used for inter-group comparisons. Effect sizes (η²) were also calculated to assess the magnitude of differences: All irradiated groups differed significantly from controls (p < 0.05) except:
  - VEGF in 810 nm: p = 0.999 (non-significant).
- Correlation analysis:
  - A negative correlation (*r* = −0.78, *p* < 0.01) was observed between fibrin network irregularity (SEM findings) and PDGF-BB/VEGF release, suggesting that structural densification at 940 nm may impede growth factor diffusion. Table 4 details statistical outcomes.

**Table 4:** Intragroup comparative analysis of PDGF-BB and VEGF-AA levels via paired t-tests in control and irradiated PRF membranes. The table summarizes statistical outcomes for platelet-derived growth factor-BB (PDGF-BB) and vascular endothelial growth factor A (VEGF-AA) concentrations, with paired comparisons revealing statistically significant differences (*p* < 0.001) in sub-groups 1, 2, and 3 following laser irradiation

Pairs- PDGF-BB	*p*
Non- irradiated- irradiated- 660 nm	0.007
Non- irradiated- irradiated- 810 nm	0.029
Non- irradiated- irradiated- 940 nm	0.019
Irradiated- 660 nm- irradiated- 810 nm	0.999
Irradiated- 660 nm- irradiated- 940 nm	< 0.001*
Irradiated- 810 nm- irradiated- 940 nm	< 0.001*
Pairs- VEGF-AA	p
Non- irradiated- irradiated- 660 nm	0.999
Non- irradiated- irradiated- 810 nm	0.999
Non- irradiated- irradiated- 940 nm	0.189
Irradiated- 660 nm- irradiated- 810 nm	0.999
Irradiated- 660 nm- irradiated- 940 nm	0.028
Irradiated- 810 nm- irradiated- 940 nm	0.067

## Discussion

### Wavelength-specific effects

This study demonstrates that PBM exerts wavelength-dependent effects on PRF, with distinct outcomes in structural remodeling and growth factor release. It is noteworthy that the proportion of smooth fibers appeared higher at 10,000× than at 5,000× across groups. This finding likely reflects the effect of magnification on classification: at higher resolution, individual fibrin filaments and their parallel alignments become more clearly discernible, whereas at lower magnification overlapping bundles and network heterogeneity are more likely to be scored as irregular or clustered. Similar magnification-dependent differences have been reported in other fibrin-based biomaterial analyses [19, 20], underscoring the importance of considering scale effects when interpreting SEM-derived morphology percentages. In this study, it was found that the 660 nm subgroup exhibited the highest VEGF and PDGF-BB secretion, consistent with prior findings that red light (600–700 nm) enhances mitochondrial cytochrome c oxidase activity, thereby stimulating ATP synthesis and growth factor production [7]. In blood-derived matrices, PBM has been reported to alter platelet activation and fibrin network organization, consistent with our findings [21, 22]. Beyond mitochondrial activation, the superiority of 660 nm may also derive from its specific chromophore absorption profile: hemoglobin and flavoproteins show higher absorption in this range, leading to increased local photochemical activity and reactive oxygen species signaling [11]. These effects can amplify downstream cellular pathways, including transcription factor activation (e.g., NF-κB, AP-1), which are involved in cytokine and growth factor release. Moreover, 660 nm irradiation may enhance platelet degranulation and leukocyte-mediated cytokine secretion, both of which are integral to PRF’s regenerative profile [23]. Collectively, these overlapping mechanisms—mitochondrial stimulation, chromophore absorption, and cell-mediated signaling—likely explain why 660 nm irradiation yielded superior biochemical outcomes compared with 810 and 940 nm.

The 810 nm (NIR spectrum) subgroup showed intermediate effects. With greater tissue penetration than red light, 810 nm primarily modulates cytochrome c oxidase and nitric oxide dissociation, enhancing mitochondrial respiration and angiogenic signaling [24]. This may explain the modest but consistent increases in PDGF-BB and VEGF release, even though the effects did not reach the magnitude of 660 nm. Importantly, the deeper penetration of 810 nm may be more relevant in in vivo contexts, where target tissues extend beyond the superficial PRF matrix.

The 940 nm (NIR spectrum) subgroup exhibited the most pronounced structural remodeling of the fibrin network but showed limited growth factor release, characterized by densely interwoven fibrin fibers with reduced pore size and overall network compaction, consistent with our quantitative SEM observations. The mechanism may involve photothermal and photochemical effects, such as fibrin network densification/compaction, which stabilize the PRF scaffold but simultaneously restrict diffusion of entrapped cytokines [9, 25, 26]. These findings are consistent with prior in vivo studies reporting that 940 nm lasers enhance bone regeneration despite modest biochemical stimulation, suggesting wavelength-specific structural benefits that complement biochemical pathways [7, 25].

In this ex vivo model, light primarily interacts with the PRF matrix of standardized thickness and hydration, so the balance of absorption/scattering differs from in vivo, where deeper tissues (mucosa, periosteum, bone) contribute to NIR energy deposition. Red (660 nm), with higher absorption by superficial chromophores (e.g., hemoglobin/flavoproteins), may favor early biochemical release within PRF, whereas NIR (810/940 nm) exhibits greater penetration and can promote matrix consolidation and deeper-tissue signaling. This distinction supports our phase-specific translational proposal, while underscoring that in vivo dosing and timing require confirmation to account for tissue-level energy distribution.

Collectively, these results underscore that red (660 nm) and near-infrared (810, 940 nm) wavelengths act through overlapping but distinct PBM pathways. Red light (600–700 nm) preferentially interacts with superficial chromophores (hemoglobin, flavoproteins) and strongly drives biochemical responses, whereas near-infrared light (800–1000 nm) penetrates deeper tissues, primarily modulating mitochondrial respiration and matrix stability through cytochrome c oxidase and water-associated chromophores. This duality explains why 660 nm optimized biochemical release, while 940 nm promoted structural integrity of the PRF scaffold, and 810 nm produced intermediate effects.

### Comparison with prior studies

The results of present study are consistent with previous reports showing wavelength-specific modulation of platelet-derived products. Bhatnagar et al. [26]. observed structural reorganization in PRF following low-level laser exposure, supporting our SEM-based observations. Similarly, Kalaivani et al. [27]. reported enhanced PDGF-BB release when PBM was combined with platelet concentrates, aligning with the elevated VEGF we observed at 660 nm. In contrast, studies using PBM have demonstrated improved scaffold density and bone healing [28, 29], which corresponds to the fibrin densification we noted at 940 nm despite lower growth factor release. These convergent findings suggest that while red light preferentially enhances biochemical signaling, NIR wavelengths contribute to structural stabilization of PRF scaffolds, highlighting the complementary roles of different spectra in regenerative dentistry. While this study highlights 940 nm’s structural benefits, it contrasts with Aboud et al. [25]., who reported enhanced bone healing with 940 nm despite minimal growth factor elevation. This discrepancy may stem from differences in experimental models: Aboud’s in vivo calvarial defect study prioritized osteoblast activation and mineralization, whereas our in vitro PRF analysis focused on fibrin architecture and cytokine kinetics. Additionally, variations in laser parameters (e.g., power density: 0.5 W/cm² here vs. 80 mW in Aboud) could explain divergent outcomes, as higher energy densities may induce thermal effects that indirectly stimulate bone formation.

Similarly, Fekrazad et al. [24]. observed improved healing with 810 nm in bone defects, a finding not fully replicated here. This underscores the influence of target tissue (soft vs. hard tissue) and biomaterial interactions on PBM efficacy. Our results emphasize that PRF’s response to PBM is uniquely shaped by its fibrin-leukocyte composition, necessitating wavelength-specific optimization distinct from other regenerative models.

## Limitations

This study provided novel insights into the photobiomodulatory effects of diode lasers on PRF. However, this study has still some limitations as follow:

### Small sample size

The study utilized 20 PRF samples derived from 10 volunteers and included *n* = 5 per group based on an a priori power estimate targeting a conservative large effect (f ≈ 0.50) at α = 0.05 with 80% power, and was additionally constrained by feasibility of autologous blood collection and lab throughput. Although post hoc evaluation using the observed omnibus tests suggested very large effects and high achieved power, these findings should be interpreted cautiously given the exploratory design and sample size. Larger, independently powered studies are warranted to refine effect estimates and enhance generalizability [14, 15]. In addition, although we restricted the donor age range (18–40 years) and applied strict health-related inclusion/exclusion criteria to minimize variability, individual differences in PRF composition (e.g., platelet and leukocyte counts, baseline cytokine levels) could still influence wavelength-specific outcomes as systemic and hematological parameters influencing PRF composition may vary across decades. Such donor-dependent variability is inherent to autologous biomaterials and may partly explain intra-group differences in growth factor release. Future studies should incorporate baseline hematological profiling and larger sample sizes to better account for these inter-individual biological differences. While the proposed 660-then-980 nm sequencing is biologically motivated and supported by prior PBM literature [30], the exact interval and dosing require prospective in vivo validation to confirm effects on scaffold–tissue integration and clinical endpoints. We present this as a hypothesis-generating framework for future trials.

### Single-timepoint ELISA analysis

Growth factor concentrations were assessed only at a single post-irradiation timepoint, precluding longitudinal evaluation of release kinetics. PRF is known to release growth factors gradually over 7–14 days, and a one-time measurement may not capture dynamic secretion profiles or delayed biochemical effects of laser irradiation. Subsequent research should incorporate multiple timepoints (e.g., days 1, 3, 7, 14) to elucidate temporal release patterns and optimize irradiation timing.

### Lack of in vivo validation

The in vitro design, while controlled, does not account for the complex in vivo microenvironment, where factors such as host immune responses, mechanical stresses, and vascularization influence PRF’s regenerative performance. For instance, 940 nm-induced fibrin densification observed here might enhance scaffold stability clinically but could also impede cellular infiltration in vivo. Translational studies in animal models or human trials are essential to confirm these findings and refine clinical protocols.

### Additional considerations

- Standardization of PRF preparation: Minor variations in centrifugation protocols or blood handling could affect PRF’s baseline architecture and growth factor content, potentially confounding laser effects.
- Laser parameter optimization: Fixed parameters (e.g., 0.5 W/cm², 4 J/cm²) were used for all wavelengths; future work should explore dose-response relationships to identify energy thresholds for maximal efficacy.

Addressing these limitations in future studies will enhance the clinical applicability of PBM-PRF combinations and advance personalized regenerative strategies in periodontics.

## Conclusion and future directions

This study elucidates the dual photobiomodulatory effects of diode laser wavelengths on PRF, demonstrating that 660 nm irradiation maximizes growth factor release (VEGF and PDGF-BB), while 940 nm irradiation enhances structural integrity through fibrin network densification. These findings underscore the critical role of wavelength selection in optimizing PRF’s regenerative potential, offering a strategic framework to tailor PBM protocols for distinct phases of periodontal healing. The 660 nm wavelength, by stimulating platelet degranulation and leukocyte activity, provides a biochemical boost essential for early-stage angiogenesis and fibroblast recruitment. In contrast, 940 nm irradiation promotes scaffold stability via fibrin realignment, which may support long-term tissue maturation and graft containment. Clinically, integrating 660 nm PBM during initial healing phases could accelerate soft tissue repair, while 940 nm application in later stages may enhance hard tissue regeneration by stabilizing the PRF matrix. A sequential therapeutic approach, combining both wavelengths, could mirror the natural healing cascade, synergizing biochemical and structural benefits.

The wavelength-specific outcomes of this study offer actionable insights for periodontal therapy:

- 660 nm: Optimal for early healing phases (inflammatory/proliferative stages), where rapid growth factor release is critical for angiogenesis and fibroblast recruitment. Clinically, this wavelength could be applied immediately post-surgery to PRF membranes placed in extraction sockets or intrabony defects.
- 940 nm: Ideal for later regenerative stages (maturation/remodeling), where structural integrity of the fibrin scaffold is paramount to support osteoblast activity and fibrin deposition. This wavelength may benefit guided bone regeneration procedures requiring stable graft containment.

A combined protocol—using 660 nm initially to boost growth factors, followed by 940 nm to stabilize the scaffold—could synergize biochemical and structural benefits, mirroring the natural healing cascade. Such an approach aligns with emerging trends in precision periodontics, where adjunctive therapies are customized to match tissue repair dynamics. Accumulating evidence indicates that red and near-infrared (NIR) PBM act through overlapping but partially distinct pathways relevant to periodontal regeneration: red light (~ 660 nm) strongly engages superficial chromophores and cytochrome-c-oxidase-linked signaling, enhancing early biochemical outputs, whereas NIR (810–980/940 nm) penetrates more deeply and is frequently associated with angiogenesis, fibroblast activity, and matrix remodeling in oral and cutaneous models [31]. Dual-wavelength clinical protocols in oral surgery (red + NIR applied peri- and post-operatively) report favorable healing trajectories, and PBM has been successfully combined with PRF in clinical settings (e.g., MRONJ), underscoring translational feasibility [32, 33]. In periodontal-relevant cell models, 660 vs. 980 nm PBM differentially modulates viability and osteogenic gene expression with repeated sessions, supporting the notion of phase-specific wavelength effects [30]. Guided by these data and our present ex vivo results, we propose a hypothesis-generating, two-stage clinical workflow when PBM is used adjunctively with PRF: (i) 660 nm immediately after PRF placement (intra-operative or early post-operative) to potentiate the initial surge of VEGF/PDGF-BB and cellular activation; (ii) 940 nm at 24–48 h (with optional repeats through day 3–7) to support scaffold consolidation and later-phase matrix remodeling while minimizing the risk of prematurely restricting early factor diffusion. This staged approach aligns with oral-surgery PBM trials that deliver immediate and early follow-up sessions for improved outcomes.

Future work should focus on three key aspects: (i) validating these ex vivo findings in in vivo periodontal models, (ii) developing standardized outcome metrics (structural and biochemical) to improve comparability across PBM–PRF studies, (iii) optimizing timing and dosing protocols for dual-wavelength PBM in clinical translation, and (iv) pairing wavelength optimization with expanded multiplex growth-factor profiling to map PBM-PRF effects across angiogenic, inflammatory, and osteogenic pathways. Addressing these priorities will accelerate the design of evidence-based protocols that enhance PRF’s regenerative capacity.

By addressing these priorities, researchers and clinicians can translate this work into evidence-based, personalized protocols that elevate PRF’s efficacy in regenerative dentistry. Another critical future step will be the development of standardized outcome metrics for PBM-PRF studies—including structural endpoints (fibrin fiber organization, porosity) and biochemical endpoints (growth factor release kinetics, normalized to PRF weight/volume). Such harmonization will enable direct comparisons across studies and facilitate robust systematic reviews and meta-analyses. This study not only advances the scientific understanding of PBM-PRF interactions but also paves the way for innovative, wavelength-driven strategies to combat periodontal tissue loss and improve patient outcomes.

## Supplementary Information

Below is the link to the electronic supplementary material.

Supplementary Material 1 (DOCX 20.7 KB)

## Data Availability

Data are contained within the article.
