# Supplementary material for: Comparative photobiomodulatory effects of 660 nm, 810 nm, and 940 nm diode lasers on platelet-rich fibrin: an ex vivo study on structural remodeling and VEGF/PDGF-BB release for enhanced periodontal healing
Source: Lasers Med Sci. 2025 Oct 20;40(1):446. doi: 10.1007/s10103-025-04688-1 (PMC12537602; doi:10.1007/s10103-025-04688-1)
Supplement: Supplementary file 1 — Supplementary Material 1 (DOCX 20.7 KB) [file 10103_2025_4688_MOESM1_ESM.docx]

**Comparative photobiomodulatory effects of 660 nm, 810 nm, and 940 nm diode lasers on platelet-rich fibrin: an ex vivo study on structural remodeling and VEGF/PDGF-BB release for enhanced periodontal healing**

**Table S1.** Full results of the inter-rater reliability analysis, including Cohen’s kappa (κ) for categorical morphology and intraclass correlation coefficients (ICC) with 95% confidence intervals for continuous metrics (fiber diameter, pore area, and porosity).

| Outcome (scale) | Metric | Estimate | 95% CI (lower-upper) | Interpretation |
| --- | --- | --- | --- | --- |
| Morphology (categorical) | Cohen’s κ | 0.0.82 | 0.74–0.90 | Excellent |
| Fiber diameter (continuous) | ICC(2,1) | 0.87 | 0.79-0.93 | Excellent |
| Pore area (continuous) | ICC(2,1) | 0.85 | 0.76-0.92 | Excellent |
| Porosity (continuous) | ICC(2,1) | 0.86 | 0.78-0.93 | Excellent |

**Table S2.** Standard Operating Procedure (SOP) for the blinded morphological analysis of SEM micrographs, used for examiner training and calibration. Includes guidelines for ROI selection, image thresholding, measurement workflows in ImageJ/Fiji, and decision rules for categorical scoring.

| Parameter | Scale/unit | Decision rule/threshold | Recorded as |
| --- | --- | --- | --- |
| Fiber morphology | Categorical (3-point) | Smooth (parallel, uniform); clustered (> 5 µm bundles); irregular (heterogeneous) | Category per ROI |
| Fiber diameter | Continuous (nm) | > 100 fibers/sample; scale calibrated in ImageJ | Mean ± SD |
| Pore area | Continuous (µm^2^) | Pores ≥ 0.02 µm^2^; measured via thresholding | Mean ± SD |
| Porosity | Continuous (%) | %Area from ROI thresholding; triplicate ROIs | Mean ± SD |

**Table S3.** Standardized evaluation protocol for the semi-quantitative and quantitative analysis of fibrin network morphology. The table outlines the key elements, scales, and units used by two independent, blinded examiners to ensure a consistent and objective analysis workflow, as defined by the written standard operating procedure (SOP).

| Element | Scale/unit |
| --- | --- |
| Magnification per sample | 5,000× and 10000× |
| ROIs per magnification | 3 non-overlapping randomized ROIs (total 6 ROIs/sample) |
| Fiber classification | Smooth/clustered/irregular (SOP decision rules) |
| Minimum fibers per sample/mag | ≥ 100 (combined across 3 ROIs) |
| Percentage per category | (Category fibers ÷ total fibers) × 100 (per sample), then group mean ± SD |
| Blinding | Two independent examiners, blinded to group allocation |
| Reliability | Cohen’s κ (categorical) and ICC(2,1) (continuous) with 95% CIs |
